# The Management of Deciduous Teeth

**Published:** 1882-06

**Authors:** S. E. Knowles


### THE MANAGEMENT OF DECIDUOUS TEETH.

BY S. E. KNOWLES, M.D., D.D.S.

Gentlemen:—I think you will all agree with me, when the statement is made, that the care of the temporary teeth is of much importance; that these organs are placed in the mouth for many desirable purposes, and that the "Great Architect of the Universe" had in view the welfare of infantile humanity when it was ordained that there should be provided a temporary set of organs of mastication, which fulfills so admirably the purposes for which they were designed. Yet, in the face of facts so apparent, how many of us, in our every-day practice, are willing to expend the energy and muscle, exercise the patience, and tolerate the annoyances necessary, in order to do what we know to be our duty, when we are called upon for professional advice regarding these teeth. All of us, I fear, must admit our weakness. It is, I regret to say, principally through ignorance of facts on our part, or a selfish and deeply-rooted dislike to serve young children, that the current belief amongst parents that the temporary teeth are unworthy of attention, has become so universal; arsenic or the forceps being the cure for all painful affections of these organs. In this paper, I shall endeavor to demonstrate that they are not solely for ornamentation, that they have many useful purposes to subserve, that they are indispensable to the health of the child, and that they bear relations to the permanent denture so intimate as to render their preservation a matter of vital importance. The masticating area ought to be sufficiently large for the perfect division of the food of the child; for at this period of life the forces of growth are in full activity, and the demand for formative material is great; if, therefore, the teeth fail to perform this function, the stomach is forced to digest imperfectly triturated food, thus laying the foundation for future dyspeptic disorder. The time and manner of development of these teeth do not properly come within the scope of this paper. It is sufficient for us to know that, as a rule, their eruption commences during the seventh month, and terminates ordinarily during the twenty-fourth. With the infantile disorders attending teething, we have little to do, their treatment being confided to the care of the general practitioner. We are, however, occasionally consulted regarding the propriety of lancing, and will therefore devote a few words to its consideration.

The usefulness of thus assisting nature in the process of erupting the milk-teeth has been denied. Without wishing to appear dogmatic, I desire to be put upon record as one who *knows*, that, under certain circumstances, the lance is the one thing needful to relieve the little one of all its pain. I have seen a child with rapid pulse, disordered digestion, sleepless and fretful to the last degree, cease its crying, and fall into refreshing slumber immediately after having had the lance passed through a sac containing serum, which was over an erupting tooth, and so large as to give distinctly the sense of fluctuation. When, as in this case, the effect is so marked and so immediate, there can be no doubt as to the value of this simple operation.

Concerning lancing, there is one thing that is not generally known, or at any rate, not generally practiced, which is the proper direction in which to cut, to relieve the tension over a molar.

The incisions are ordinarily made over the middle of the tooth, in a line with the curvature of the arch, and again across the center at right angles. A much better method is to make one cut from the outer back to the inner front cusp, and another from the inner back to the outer front cusp; by so doing, the incisions are made over the most prominent portions of the coming tooth, and the edges of the wound are less liable to adhere to each other and cicatrize.

There are many anomalous conditions found in connection with teeth of the first dentition; the teeth may vary in number; cases are on record in which there were more than twenty teeth. They may present the true supernumerary form, or may be so nearly like the normal type as to make it impossible to designate the supernumerary teeth. There are also cases presenting less than the normal number; instances of twin or triplet teeth have also been observed.

I have in my collection a tooth mass with three distinct roots ranged side by side, which occupied the places of the central and lateral incisors, but were without crowns when first seen by me. There are also slight irregularities in the arrangement of the teeth in the arch. In nearly every instance these unnatural conditions are in connection with the incisor teeth.

A case is recorded by Tomes, in which no temporary teeth were ever erupted, the germs probably never having been formed. The curvature of the primitive arches corresponds almost exactly with the curvature of the teeth of replacement; this fact would seem to plainly indicate the importance of having the temporary teeth kept with unbroken arches, until the first permanent molars are well in position, owing to the well-known disposition of these permanent teeth to move forward, if there should be room for such movement. The members of the temporary denture, as you are well aware, differ from those of the permanent in size, form, and density, the temporary teeth being individually smaller than their successors, and therefore collectively occupy less space.

This arrangement would be almost certain to produce crowded arches, were it not for the fact that the milk-molars occupy more space than the bicuspid teeth, and furnish the room demanded by the permanent teeth. In the management of the teeth of the first dentition, much care is necessary to prevent the irregular arrangement of the permanent teeth. The temporary incisor teeth may be extracted at almost any time, without fear of producing such condition; but an error is often committed by extracting the temporary canines, in order to allow a sufficient space for the free eruption of the permanent lateral incisors.

This is the point to which too much attention cannot be paid; for although relief is afforded for the time being, the bicuspids and laterals are thus often allowed to approximate too closely, force the permanent canines too far forward, and to produce a deformity difficult of correction, because of the necessity for changing the articulation of nearly all of the permanent teeth. While a majority of the temporary teeth are still in the mouth, an irregularity of articulation of the permanent incisors is of frequent occurrence, and it is important that this condition should be recognized early, and immediately abated; first, because it is so easy to correct before their full eruption is completed; secondly, because of the undue defacement of the cutting edges of the teeth; and lastly, because of the minor evils arising from continued distortion of the arches. The second temporary molars ought in every instance to be retained in position, if possible, until the six-year molars are erupted far enough to articulate with each other, otherwise they will move too far forward, and contract the space which should be retained for the molar and bicuspid teeth. Even dead second temporary molars ought to be kept in the mouth until this time, unless it should have become impossible to avoid their extraction on account of intractable alveolar abscess, or other sufficient reason. In case of the pulps having become devitalized, the teeth ought to be extracted after the completion of the full eruption of the first permanent molars, and before the time for the eruption of the bicuspids. It is a very common error on the part of parents to regard the first permanent molars as belonging to the first dentition, and, as a consequence, many of these teeth are brought to us which are past salvation. This mistake is quite natural, as they appear so early in life, and do not replace any of the milk-teeth.

To one imperfectly informed about dental matters, such an inference is quite natural, and shows the need of proper instruction upon this point.

The first temporary molars are of comparatively little importance, certainly not more so than are the temporary incisors. The retention of roots of the temporary teeth is a frequent cause of irregularities in the arrangement of the teeth of the second dentition.

As a rule, all crownless roots should be extracted. Another point in this connection: dead temporary teeth should be removed from the mouth sometime before their individual successors are due, because most likely their roots have not been shortened by absorption.

In the extraction of either of the molar teeth, the fact must be borne in mind that the imperfectly developed bicuspids are in close relation with their roots, and the utmost care is to be exercised that they may not be disturbed. The normal process of absorption is a manifestation of vital force, and is found to affect vital structures only. It is not a progressive breaking down of the old structures; but there is found to be a building up and breaking down going on at the same time, in different parts of the same root, or a building and removing alternately.

When teeth are pulpless, the cementum alone offers a tissue in which true normal absorption can take place; the wasting of the dentine being very slow, and due entirely to chemical action. Operations upon the temporary teeth, having for their object their salvation, are in order at a very early age, frequently as early as two and one-half years. It will not be necessary to enlarge upon the difficulties to be encountered; I have, however, in two instances succeeded in filling molars with amalgam at this time of life; one child is now six, and the other seven years of age, and the teeth have thus far been preserved.

The period demanding the majority of operations on the temporary teeth is between the fourth and ninth years, and during this time the child is usually sufficiently manageable. I am free to confess, however, that the little ones frequently tax our patience severely; at times, beyond the limits of endurance. The preparation of the temporary teeth for the reception of appropriate filling materials does not differ much from that of the permanent teeth, but it should be remembered that the pulps are proportionately larger, and relatively nearer the surface.

If by accident, disease, or other cause, the pulp should be found exposed, capping and temporary filling are in order; then, in case of death of the pulp, and consequent alveolar abscess, the tooth is in proper condition for tapping, to supply a vent for the gases and fluids which have accumulated. This operation is, as a rule, better practice than is an attempt to fill the root canals.

In the treatment of decay on the proximal surfaces, the free use of the separating disk is to be recommended, especially for decay of the molars; the spaces should be distinctly flaring from the neck toward the grinding surface, and from the buccal toward the lingual and palatine sides; this provides somewhat against its recurrence, furnishes surfaces readily cleansed, and of easy inspection. For decay in this locality, the plastic filling materials are in order, for obvious reasons, especially the gutta-percha and amalgam preparations; for simple crown cavities, however, whenever possible to properly use it, there is nothing like our sheet-anchor, gold. The rubber dam is especially important in operations on the milk-teeth, as the saliva is so abundantly secreted; these teeth are also of such texture as to render imperfect operations comparatively valueless; extraordinary care should, therefore, be used to make these operations even more perfect than similar ones performed on the permanent teeth.

Inasmuch as the object of this paper has been more to provoke discussion than to comprehend fully the subject in hand, I have contented myself with alluding thus briefly to a few of the salient points, which I trust will, with others omitted, be more ably set forth and more fully amplified by the members present.

### DISCUSSION.

DR. W. J. PRATHER. I fully agree with the essayest in the position taken with reference to deciduous teeth. I have noticed the evil effects of their neglect for years past. And in fact, as far back as nineteen years ago, I filled deciduous teeth, and kept them in perfect condition until the time for their removal.

When my son's teeth had attained their full length, a black line showed itself at the margin of the gum. I neglected it, and in a short time the teeth were destroyed, and I had to remove them.

With my next child, at one year old the line made its appearance, and I removed that, and polished the teeth by the use of the engine. She is now five years old, and the teeth have remained good. I am confident, if they had been neglected, they would have been lost, as were those of her brother.

Her brother having had his teeth removed when he was so very young, when the other teeth made their appearance, they came in rather irregular.

Dr. S. E. Knowles. I have a specimen of a perfectly normal absorption going on after the tooth had been filled. It is a first temporary molar tooth. That filling was in between two and three years.—*Transactions Cal. State Dental Association.*

